# Endovascular Repair of Persistent Sciatic Artery With Limb Ischemia: A Wrong Choice?

**DOI:** 10.3389/fsurg.2020.582753

**Published:** 2020-11-16

**Authors:** Liming Deng, Zhihe Deng, Kaiyu Chen, Ziyan Chen, Gang Chen, Guozuo Xiong

**Affiliations:** ^1^Department of Vascular Surgery, The Second Affiliated Hospital of University of South China, Hengyang, China; ^2^Department of Hepatobiliary Surgery, The First Affiliated Hospital of Wenzhou Medical University, Wenzhou, China

**Keywords:** persistent sciatic artery, thromboembolism, endovascular repair, malformation, limb ischemia

## Abstract

**Objectives:** Persistent sciatic artery (PSA) is a rare congenital malformation that could lead to serious complications such as lower extremity ischemiais. We report the treatment of a PSA patient combined with limb ischemia.

**Methods:** A 64-year-old man was admitted to the hospital for intermittent claudication of the right lower limb. The right ankle–brachial index (ABI) was 0.5. Computed tomography angiography (CTA) revealed the presence of an incomplete PSA with an absence superficial femoral artery. We performed catheter-directed thrombolysis (CDT) and stenting placement for the patient.

**Results:** The vessels were successfully opened, and the claudication was resolved. However, half a year after the operation, the right PSA of the patient was occluded.

**Conclusion:** The etiology, pathophysiology, and anatomic factors should be considered in the treatment of PSA. Endovascular treatment, bypass surgery, and drug therapy should be balanced. Improper choice of any treatment regimen may result in poor prognosis.

## Introduction

Persistent sciatic artery (PSA) is a rare vascular congenital malformation, firstly described in 1832 by Green (1). The incidence of PSA is estimated at 0.01–0.06% of the population (2). Patients are asymptomatic in the majority of cases. However, it can cause complications, including acute and chronic limb ischemia and aneurysm formation. We reported a case of limb ischemia patient with a concomitant PSA treated by endovascular repair.

## Case Presentation

**Figure 1:**
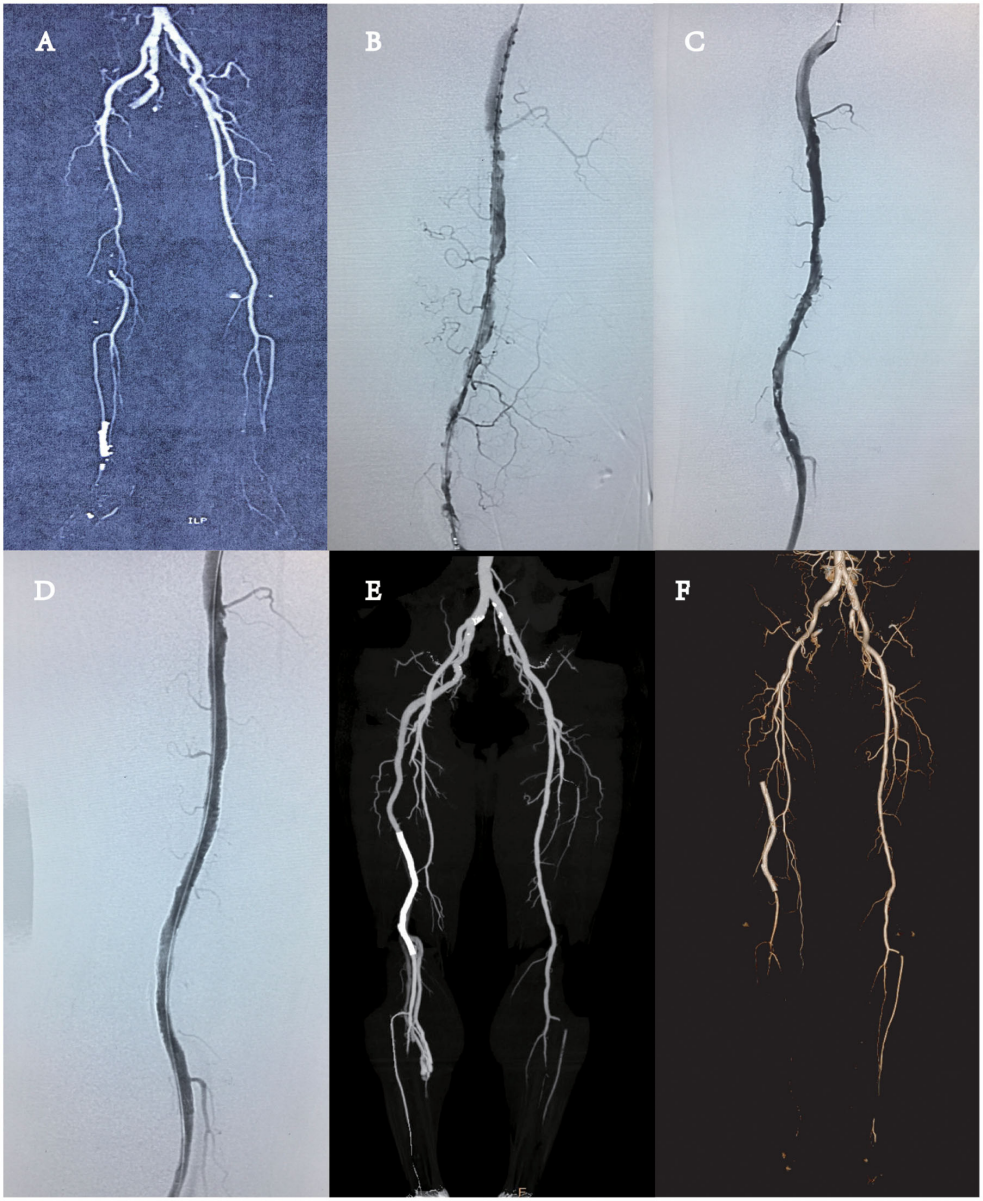
**(A)** Computed tomography angiography (CTA) showed the presence of complete persistent sciatic artery (PSA) with an incompletely developed superficial femoral artery (SFA) (type 2A PSA), without any aneurysmal change, and the complete obstruction of near-end popliteal artery by thrombus. **(B)** DSA revealed the obstruction of the remote of PSA and popliteal artery by thrombus. **(C)** One day of thrombolysis digital subtraction angiography (DSA) examination revealed partial dissolution of the thrombus in the blood vessels and multiple stenoses in the lumen, and advanced thrombosis was considered. **(D)** DSA revealed the vascular stent patency and no stenosis and thrombosis. **(E)** CTA revealed the patency of the whole PSA. There was no thrombosis or stenosis in the vascular stent. **(F)** CTA revealed that thrombosis in the vascular stent and the vessel was occluded.

A 64-year-old male patient with no previous history of trauma was admitted to the hospital for intermittent claudication of the right lower limb for more than 1 month. The patient did not have resting pain or numbness in the lower extremities, and the claudication distance was about 100 m. The clinical history of acute myocardial infarction was evidenced by percutaneous coronary intervention (PCI) *via* the radial approach 6 months ago, and one stent was implanted. After the operation, the patient took aspirin and clopidogrel regularly. The patient had a history of smoking and drinking but did not have hypertension, diabetes, and family genetic disease. Physical examination revealed that the right common femoral pulse was palpated, but popliteal pulse and anterior tibial arteries were not palpable. The right ankle–brachial index (ABI) was 0.5. The preoperative Rutherford classification was level 3. Computed tomography angiography (CTA) revealed the absence of superficial femoral artery (SFA) (typeIIb PSA). The thrombus in the remote of PSA and near-end popliteal artery were also noted, and the distal arteries below the knee were not obstructed (Figure 1A).

There was no external compression on the vessel. In this way, two treatment plans could be taken into consideration. Plan A: Choose aspirin and clopidogrel antithrombotic therapy with regular review by ultrasonography or CTA until claudication becomes more serious and an operation would be undertaken. Plan B: Choose endovascular treatment or femoropopliteal bypass surgery. We informed the patient of the advantages and disadvantages of the two treatment plans. Open surgery is more invasive, but the effect of surgery is better after the establishment of the bypass vessel. Endovascular treatment is minimally invasive, but stent implantation may be required, and the long-term patency rate is slightly lower. The patient and their families decided to take endovascular repair.

The patient underwent percutaneous arteriography and catheter-directed thrombolysis (CDT) of the PSA using infusion catheter (Merit Medical, Utah, USA) and urokinase (Figure 1B). One day after thrombolysis with 300,000 units of urokinase, the blood examination showed that the fibrinogen content was 1.2 g/L. Digital subtraction angiography (DSA) revealed partial thrombolysis, multiple stenoses, and an old residual thrombus in the lumen (Figure 1C). The thrombus was covered by implantation of a peripheral vascular stent (LifeStent, 5 × 170 mm, Bard, USA), the whole segment of the artery with the thrombus was covered (Figure 1D), and the distal end of the stent did not come into the popliteal artery at the knee joint. The puncture site of the left femoral artery was closed with ProGlide (Abbott Vascular, CA, USA). The right ABI increased to 1.0 from 0.5. The patient was given antiplatelet agent (aspirin and clopidogrel) after surgery and was discharged 5 days after the endovascular repair. The postoperative recovery was favorable, and the patient was discharged without any complications. CTA revealed the patency of the whole PSA and iliofemoral artery system. No residual thrombus was noted. There was no thrombosis or stenosis in the vascular stent (Figure 1E). Half a year after the operation, the patient had claudication again. CTA showed that the right PSA distal was occluded from the pelvic level (Figure 1F). We recommend that the patient should take femoropopliteal bypass surgery to rebuild blood flow and relieve lower extremity ischemia. The drug regimen could be replaced by aspirin antithrombotic and rivaroxaban anticoagulant therapy. Somehow the patient refused the operation and chose the medication regimen. Up to now, the patient's symptoms still remained. The patient was recommended to return to the hospital for further consultation every 3 months.

The study was approved by the ethics committee of the Second Affiliated Hospital of University of South China, and informed consent was obtained from the patient by telephone.

## Discussion

There were ~200 cases of PSA having been reported. PSA is a continuation of the internal iliac artery in early embryonic development, majorly supplying the vascular of the lower limb bud. There are five different types of PSA (2). According to the presence of SFA, TypeII can be subtyped into typeIIa (presence of incomplete SFA) and typeIIb (absence of SFA). In this patient, PSA was typeIIb. In spite of the low incidence of PSA, it is associated with various complications such as atherosclerosis and aneurysms, which can result in embolization of the distal artery or thrombosis in the PSA. An aneurysm is one of the most frequent complications of PSA, with an incidence up to 60%. Lower limb ischemia is mostly caused by thromboembolism within aneurysms. In this case, we did not find an aneurysm. The reason for the lower limb thrombosis may be caused by atherosclerotic or cardiogenic factors. Unfortunately, we only took arteriosclerosis into consideration and ignored heart factors, so we chose the corresponding follow-up treatment plan at that time, which may be the possible cause of the recurrence of PSA obstruction half a year after surgery.

PSA is often asymptomatic, and the diagnosis of PSA relies on physical examination. The Cowie's sign of PSA is that there is the presence of distal pulsation without femoral artery pulsation. CTA is the gold standard for diagnosis, and ultrasound can diagnose patients with suspected PSA (3). In this case, Cowie's sign was negative due to the thrombosis in the distal artery.

The treatment of PSA depends on the symptoms, vascular anatomy of the PSA and the iliofemoral system, and the presence of concurrent vascular occlusive disease and aneurysm (4, 5). Asymptomatic patients require close follow-up. Wang et al. (6) reported that a 15-year-old girl has bilateral PSAs complicated with chronic lower limb ischemia. However, they did not take any intervention, only kept surveillance with duplex ultrasonography (6). Symptomatic patient's treatment should be based on the presence of complications and classification of PSA. Incomplete PSA with the aneurysmal change, with or without the vascular occlusive disease, can be obliterated by ligation, resection, embolization, or endovascular stent graft (7–10). Vascular reconstruction can be performed by the femoropopliteal bypass, lipoplatin trans obturator bypass, or interposition bypass (11). Wu et al. (12) reported a case treated with femoral to popliteal bypass with expanded polytetrafluoroethylene (ePTFE) graft (12). Non-aneurysmal PSA with symptomatic thromboembolism can be managed with embolectomy either by endovascular intervention or open surgical approach.

In our case, the treatment strategy for PSA is CDT and stent implantation, which completely cover the vessels in the restenosis and thrombosis segment and make the occluded vessels open. However, half a year after the operation, the patient's right PSA thrombus formed again and occluded the vessels. Taking the whole treatment process into consideration, the treatment plan of endovascular treatment for the patient was a wrong choice. The claudication distance of the patient was <100 m, and the patient had the requirement of improving the quality of life. But the patient opposed the bypass surgery. Therefore, there was no mistake in choosing endovascular treatment. However, the patient experienced vascular occlusion again after treatment, which may be due to the imperfect postoperative drug treatment regimen. The thrombogenic stage of PSA in patients is most likely due to anatomic malformations of lower limb vessels and abnormal blood flow due to cardiac factors. Antiplatelet and anticoagulant therapy should be chosen as drug therapy. Bypass surgery is an option, but the risk of reobstruction is high. Research reports showed that low-dose rivaroxaban plus aspirin reduced major adverse cardiovascular and limb events when compared with aspirin alone (13). During cardiac remodeling after myocardial infarction, anticoagulant and antithrombotic therapies are appropriate. Patients choose anticoagulant and antithrombotic drug therapy instead of surgical treatment after vascular occlusion again, and the symptoms do not get worse, which indirectly proves the importance of drug treatment selection.

## Conclusion

The etiology, pathophysiology, and anatomic factors should be considered in the treatment of PSA. Endovascular treatment, bypass surgery, and drug therapy should be balanced. Improper choice of any treatment regimen may result in poor prognosis.

## Data Availability Statement

The raw data supporting the conclusions of this article will be made available by the authors, without undue reservation.

## Ethics Statement

The studies involving human participants were reviewed and approved by the Institutional Ethics Committee of the Second Affiliated Hospital of University of South China. Written informed consent to participate in this study was provided by the participants' legal guardian/next of kin. Written informed consent was obtained from the individual(s) for the publication of any potentially identifiable images or data included in this article.

## Author Contributions

LD, GC, and GX conceptualized and designed the study, had full access to all the data in the study and had responsibility for the integrity of the data, the accuracy of the analyses, and the final decision to submit the manuscript for publication. ZD, KC, and ZC collected the data and performed the analysis. LD and ZD drafted the initial version of the manuscript. All authors contributed to the interpretation of the results, critically reviewed many revisions of the manuscript, and contributed important intellectual content.

## Conflict of Interest

The authors declare that the research was conducted in the absence of any commercial or financial relationships that could be construed as a potential conflict of interest.
